# Correction to “Genotype–phenotype correlations in individuals with pathogenic *RERE* variants”

**DOI:** 10.1155/humu/9821512

**Published:** 2025-12-19

V. K. Jordan, B. Fregeau, X. Ge, et al., “Genotype–phenotype correlations in individuals with pathogenic *RERE* variants,” *Human Mutation* 2018, no. 39, 666–675, https://doi.org/10.1002/humu.23400.

In the article titled “Genotype–phenotype correlations in individuals with pathogenic *RERE* variants,” Table 1 contained an error in Row S3. The corrected data are now reflected in the revised Table 1 as follows.

**Table 1:** *RERE* deletions and sequence variants identified in Subjects 1–9.

**Subject**	**Deletions and sequence variants affecting *RERE*** ^**a**^	**Inheritance**	**PolyPhen-2 (HumVar)**	**SIFT**	**MutationTaster** ^**b**^	**Allele present in the ExAC browser or gnomAD?**
S1	Minimum deletion (hg19) chr1:8,509,888‐8,803,072 Maximum deletion (hg19) chr1:8,497,191‐8,813,784	N/D	N/A	N/A	N/A	N/A
S2	c.248dupA p.(Ser84Valfs∗4)	De novo	N/A	N/A	Disease causing	No
S3	c.4300T>C p.(Ser1434Pro)	De novo	Probably damaging	Damaging	Disease causing	No
S4	c.4303C>T p.(His1435Tyr)	De novo	Possibly damaging	Damaging	Disease causing	No
S5	c.4304A>G p.(His1435Arg)	De novo	Probably damaging	Damaging	Disease causing	No
S6	c.3292C>G p.(Leu1098Val)	De novo	Possibly damaging	Tolerated	Disease causing	No
	c.4304A>T p.(His1435Leu)	De novo	Probably damaging	Damaging	Disease causing	No
S7	c.4313_4318dupTCCACC p.(Leu1438_His1439dup)	De novo	N/A	N/A	Polymorphism	No
S8	c.4313_4318dupTCCACC p.(Leu1438_His1439dup)	De novo	N/A	N/A	Polymorphism	No
S9	c.4391A>G p.(His1464Arg)	De novo	Benign	Tolerated	Disease causing	No

Abbreviations: N/A, not applicable; N/D, not determined due to a lack of parental samples.

^a^Based on *RERE* transcript variant 1[NM_012102.3]. Nucleotide (cDNA) numbering uses +1 as the A of the ATG translation initiation codon in the reference sequence, with the initiation codon as Codon 1.

^b^MutationTaster classifies all variants as either “disease causing” or “polymorphism.” These classifications do not indicate that the variant has been shown to cause disease or that the variant is found at a frequency ≥ 1%.

We apologize for this error.

